# Multi-Modal Neuroimaging Neural Network-Based Feature Detection for Diagnosis of Alzheimer’s Disease

**DOI:** 10.3389/fnagi.2022.911220

**Published:** 2022-05-16

**Authors:** Xianglian Meng, Junlong Liu, Xiang Fan, Chenyuan Bian, Qingpeng Wei, Ziwei Wang, Wenjie Liu, Zhuqing Jiao

**Affiliations:** ^1^School of Computer Information and Engineering, Changzhou Institute of Technology, Changzhou, China; ^2^Shandong Provincial Key Laboratory of Digital Medicine and Computer-Assisted Surgery, Affiliated Hospital of Qingdao University, Qingdao, China; ^3^School of Computer Science and Artificial Intelligence, Changzhou University, Changzhou, China

**Keywords:** multi-modal, LassoNet, resting state functional magnetic resonance imaging, diffusion tensor imaging, feature detection

## Abstract

Alzheimer’s disease (AD) is a neurodegenerative brain disease, and it is challenging to mine features that distinguish AD and healthy control (HC) from multiple datasets. Brain network modeling technology in AD using single-modal images often lacks supplementary information regarding multi-source resolution and has poor spatiotemporal sensitivity. In this study, we proposed a novel multi-modal LassoNet framework with a neural network for AD-related feature detection and classification. Specifically, data including two modalities of resting-state functional magnetic resonance imaging (rs-fMRI) and diffusion tensor imaging (DTI) were adopted for predicting pathological brain areas related to AD. The results of 10 repeated experiments and validation experiments in three groups prove that our proposed framework outperforms well in classification performance, generalization, and reproducibility. Also, we found discriminative brain regions, such as Hippocampus, Frontal_Inf_Orb_L, Parietal_Sup_L, Putamen_L, Fusiform_R, etc. These discoveries provide a novel method for AD research, and the experimental study demonstrates that the framework will further improve our understanding of the mechanisms underlying the development of AD.

## Introduction

Alzheimer’s disease (AD) is a neurodegenerative brain disease that leads to the damage and death of brain nerve cells in disease progression. It destroys people’s memory, learning, language, cognition, life, and other abilities, and seriously affects the quality of life of patients and families (Zhang and Wang, 2015; Lam et al., 2021; Lim et al., 2021). AD risk is also greater later in life for people with cardiovascular disease, high blood pressure, and diabetes. The Alzheimer’s Association published a “2021 Alzheimer’s Disease Facts and Figures,” reporting a significant increase in AD deaths worldwide due to the COVID-19 pandemic. According to the clinical symptoms of patients, Alzheimer’s disease is divided into a normal state (normal control, NC), mild cognitive impairment (mild cognitive impairment, MCI) state, and diseased AD state. MCI manifests as a decline in memory and thinking abilities at a rate greater than the decline in perception caused by normal aging, but this decline does not interfere with normal social interaction and work. However, patients with MCI have a high probability of further deterioration to AD (Zhang et al., 2016; Wang et al., 2017). It is currently difficult to distinguish MCI from memory decline due to normal aging, and MCI involves very subtle brain changes. Therefore, the early diagnosis of MCI/AD is extremely challenging (Davis et al., 2018; Wang et al., 2018; Zhang et al., 2018; Potashman et al., 2021).

Magnetic resonance imaging (MRI) has become a hot spot in the field of AD and MCI disease research due to its non-invasiveness, multi-sequence imaging, high resolution, and strong repeatability (Zhang Y.-D. et al., 2014; Zhang et al., 2015a). Resting-state functional MRI (rs-fMRI) and MRI diffusion tensor imaging (DTI) are imaging techniques that can study brain mechanisms from the perspective of human brain functional connectivity and structural connectivity. They provide imaging evidence for the pathological studies on AD and MCI. Many studies have found the network structure related to the resting state in the cerebral cortex, which covers the brain regions that show a decline in metabolic function in the early stages of AD, including the posterior cingulate cortex and the internal parietal region (Choo et al., 2010; Hu et al., 2014; Zhang et al., 2015b; Shim et al., 2017; Wang et al., 2021). Neuroimaging data from a single modality usually can only reflect part of the brain characteristics, but many current research studies show that the fusion of information from multiple imaging modalities can reflect the brain activity mechanism more comprehensively (Zhang Q et al., 2014; Zhang and Shi, 2020; Lei et al., 2021; Jiao et al., 2022).

Functional MRI quantifies the temporal correlation between brain regions by detecting the blood oxygen level dependence (BOLD) in the human brain (Zhang and Shi, 2020; Wang et al., 2017), while DTI can track the spatial correlation of white matter fiber tracts by exploiting the kinetic mechanism of water molecule diffusion. Combining the spatiotemporal high-resolution information reflected by fMRI and DTI can comprehensively describe biological brain characteristics from a spatiotemporal perspective and improve the accuracy of brain network modeling, which is of great scientific significance for studying the neurophysiological mechanisms of AD/MCI diseases (Dyrba et al., 2015; Aderghal et al., 2020; Xu et al., 2021). Wee et al. considered the information regarding the complementary features of multiple imaging techniques, integrated multi-modal information from DTI and rs-fMRI, and used multi-kernel support vector machines to build a classifier for the study of disease classification and early prediction of MCI (Dai et al., 2019). Schonberg et al. used fMRI to define the regions of interest for DTI, providing a more comprehensive and functionally relevant white matter mapping map for preoperative preparation of brain tumors (Schonberg et al., 2006). Qi et al. propose a framework that combines DTI and fMRI multimodal imaging data to accurately identify potential neurological markers responsible for working memory deficits (Qi et al., 2018). Li et al. integrated the image information of rs-fMRI and DTI into a Lasso modeling framework for the accurate diagnosis of brain network lesions in early AD, further demonstrating that fusion of multi-modal information can effectively identify brain network features (Li et al., 2020). The above-mentioned finding proves that compared with single-modal data, more valuable features can be obtained by using multi-modal data. The multi-modal fusion method may further improve the recognition accuracy of AD/MCI (Zhang et al., 2015b; Wang et al., 2016; Mak et al., 2017).

**FIGURE 1:**
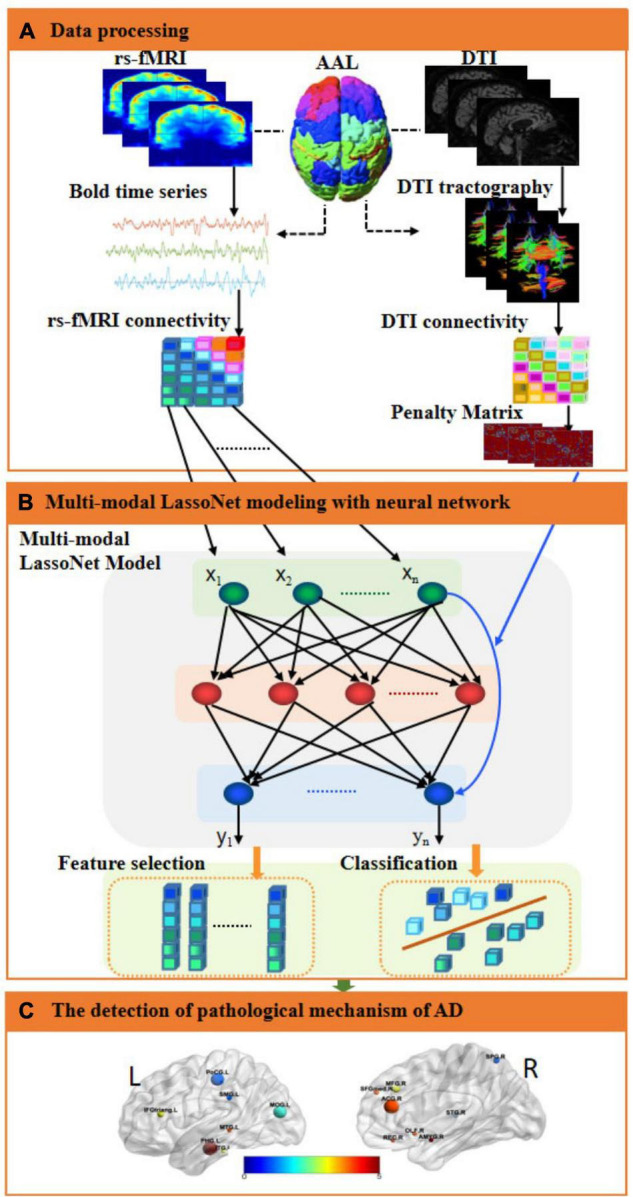
An illustration of the proposed multi-modal framework for AD. **(A)** Data processing. The fMRI and DTI images were preprocessed, and then the regions of interest were extracted as fMRI and DTI features through the AAL template, and the corresponding brain networks of fMRI and DTI were obtained, respectively. Then, computed the inverse proportional function of the structural brain network as a penalty matrix. **(B)** Multi-modal LassoNet Modeling with a neural network. We constructed a multi-modal network framework for feature selection and classification based on the LassoNet model. It consisted of residual connection and an arbitrary feed-forward neural network. The input to the network was the fMRI feature information. The penalty matrix was introduced to the residual connection to sparse features. **(C)** The detection of the pathological mechanism of AD. We visualized brain regions for selected features to analyze the affected discriminative brain regions.

In multi-modal neuroimaging analysis, since the features extracted from the original images tend to have higher dimensionality, only a few clinical samples contain complete multi-modal data, which will produce the curse of dimensionality. Therefore, we propose a neural network framework with Lasso regression for multi-modal image feature extraction and classification. Figure 1 illustrates the neural network framework of multi-modal neuroimaging for Alzheimer’s disease.

## Materials and Methods

### Data Processing

The images of 85 subjects (33 healthy control, 29 early mild cognitive impairment, and 23 AD) were downloaded from the Alzheimer’s Disease Neuroimaging Initiative (ADNI^1^), including rs-fMRI and DTI. All neuroimaging data were obtained using a SIEMENS 3T MRI scanner. For the rs-fMRI images, the echo time (TE), the repetition time (TR), the flip angle, the slice thickness, and the time points were set as 30.0 ms, 3.0 s, 90, 3.4 mm, and 197, respectively. For the DTI data, the gradient directions, the echo time, the repetition time, the flip angle, and the voxel size were set as 30, 95 ms, 12.4 s, 90, and 2×2×2 ms^3^. For the T1 images, the TE, TR, flip angle, the slice thickness, and the T1 time were set as 3.0 ms, 2.3 s, 9.0, 1.0 mm, and 900 ms, and the collection plane was SAGITTAL. The Table 1 showed the significant differences among the three groups in terms of gender (*p < 0.001*), age (*p < 0.001*), MMSE (*p < 0.001*), and EDU (*p < 0.001*) by *t*-test.

**TABLE 1:** Participant characteristics.

Subjects	HC	EMCI	AD	*P*
Number	33	29	23	
Gender (M/F)	12/21	14/15	14/9	<0.001
Age (Mean ± sd)	73.88 ± 7.15	74.52 ± 7.30	74.34 ± 8.14	<0.001
MMSE (Mean ± sd)	29.15 ± 1.13	28.52 ± 1.45	21.78 ± 1.89	<0.001
EDU (Mean ± sd)	16.55 ± 2.34	16.31 ± 2.56	14.96 ± 1.90	<0.001

*HC, healthy control; EMCI, early mild cognitive impairment; AD, Alzheimer’s disease; MMSE, Mini-mental status examination; M/F, male/female; Edu, education; sd, standard deviation.*

### Data Acquisition

The rs-fMRI images were processed using SPM12^2^ (Han and Glenn, 2018) and DPARBI 6.1^3^ (Yan et al., 2016) as follows: (1) The raw DICOM files were converted to NIFITI format. (2) The first 10 time series nodes of each individual subject were removed manually to avoid the magnetic field inhomogeneity problem caused by the startup of the scanner and the influence of the discomfort of the subject’s initial state on the results. (3) The interslice scan times were corrected to the same time point. (4) Images with head movement beyond 2.5 mm translation or 2.5-degree rotation were removed to correct head movement during scanning. (5) The head motion, white matter signal, and cerebrospinal fluid signal were set as the main noise covariates to reduce the influence of noisy covariate signals on scan results and reduce biological artifacts. (6) Different morphological brains were standardized to the same standard template and were registered to T1 images. (7) The 4 × 4 × 4 mm^3^ Gaussian kernel was applied for spatial smoothing to reduce spatial noise. (8) The linear trend was removed, and 0.01–0.1 Hz filtering was applied to reduce the interference due to low-frequency and high-frequency noise. The automated anatomical labeling (AAL; Tzourio-Mazoyer et al., 2002) atlas was applied to segment the brain into 90 regions, and the time series of BOLD signals were extracted.

The DTI data were processed using FSL^4^ (Woolrich et al., 2009), PANDA^5^ (Abbasi et al., 2021), and MRIcron (NITRC: MRIcron: Tool/Resource Info) software in Ubuntu18.04 as follows: (1) The raw DICOM files were converted to NIFITI format (*.nii.gz). (2) The brain templates were estimated based on non-diffusion-weighted b0 images using the bet command. (3) The non-brain space was removed using the fslroi command and eddy current correction. (4) The diffusion tensor metric was calculated using the dtifit command. (5) Deterministic white matter tract in the brain was tracked using the dti_recon and dti_tracker commands. (6) A part of the skull tissue in the T1 images was removed using the bet command. (7) The fractional anisotropy (FA) value of each subject was registered to its corresponding T1 image using the flirt command of FSL. When DTI images were registered with other images, DTI data causing significant deformities were removed. It should be noted that the DTI images and rs-fMRI images were registered with the same T1 imaging.

### Multi-Modal LassoNet Framework Construction

The rs-fMRI functional brain networks can measure temporal correlations between anatomically segmented brain regions; DTI-based structural brain networks can characterize and track spatial white matter tracts in the brain. Herein, it is considered to unify the multi-modal image information of rs-fMRI and DTI in a brain network modeling framework, combining the respective advantages of the two modalities, which can describe the dynamic mechanism of the brain network from the perspective of time and space, and realize the construction of the brain network model.

After preprocessing of fMRI images, we obtained 187 time series (BOLD signal) of 85 participants, and there were 90 ROIs in each image. Let us assume that we have *n* participants and *i* ROIs. We explored a multi-modal network framework for feature selection and classification based on the LassoNet (Yan and Bien, 2017; Chen et al., 2019). For *n* participant, we assumed that the fMRI time series of the *i*-th ROI was *x*_*i*_={*x*_1*i*_,*x*_2*i*_,..*x*_*di*_} ∈ *R^n×*d*^*,(*i* = 185), where *d* was the number of time points. Our goal was to find the best function f*(*x*_*i*_) for predicting *y_i* (the type of Alzheimer’s diagnosis). As the problem of learning f*(*x*_*i*_) is non-parametric, we assumed that there was no linear or quadratic restriction. The multi-modal network consisted of two parts: residual connection and arbitrary feed-forward neural network. The penalty was introduced to the residual connection to sparse features. We define *G* to be the class of residual feed-forward neural networks:


(1) $$G=\left\{f\equiv f_{\theta },w:x⟼\theta^{T}\,X+g\,w\,\left(X\right)\right\}$$

where *gW*(*X*) denotes a feed-forward network with weights *W*, *W*^(1)^ ∈ ℝ^*d*×*K*^ represents the weights in the first hidden layer, and θϵ^*Rd*^ represents the weights in the residual layer.

Let *L* be the empirical loss^2^ on the training set with fMRI time series, then *L* is defined as Equation 2.


(2) $$L\,\left(\theta ,W\right)=\frac{1}{n}\,\sum_{i=1}^{n}\ell \,\left(f_{\theta },w\,\left(x_{i}\right),{𝒴}_{i}\right)$$

where *W* is the weight of the first hidden part; θ is the weight of the residual part; *n* is the number of participants as training observations size, and ℓ is the loss function. The LassoNet model objective function is defined as Equation 3.


$$\begin{array}{c} m\,i\,n\,i\,m\,i\,z\,e\\ \theta ,W \end{array}\,L\,\left(\theta ,W\right)+\lambda \,{||\theta ||}_{1}\,s\,u\,b\,j\,e\,c\,t\,t\,o\,||W_{i}^{\left(1\right)}||\leq M\,\left|\theta_{i}\right|,$$

(3) i=1,⋯⁢d

where $W_{i}^{\left(1\right)}$ is the weight of feature *i* and *d* is the data dimension.

The coupling strength of human brain functional connectivity and structural connectivity is closely related to the brain excitation process, and stronger structural brain connectivity is likely to lead to the enhancement of corresponding functional connectivity. Here, we introduced a parameter named the punishment factor to improve the LassoNet model. The punishment matrix of each DTI image is defined as the inverse proportional function of structural brain networks (Equation 4).


(4) $$D_{j\,i}=e^{-\frac{\rho_{j\,i}^{2}}{\sigma }}$$

where ρ_*ji*_ is the FA information between *j-*th brain region and *i-*th brain region in the DTI network, and σ is the mean of the standard deviation of all elements in the structural brain network of all participants. Equation 4 is used to penalize the estimated connection strength value between the *j*-th ROI and the *i*-th ROI.

Since each participant had a corresponding DTI structure network information *D*, we calculated the max feature λ_*max*_ of each *D* using Equation 5.


(5) $$\left(\lambda_{a}\,E-D\right)\,x=0$$

where λ_*max*_=max(λ_*a*_), *E* is the unity matrix, and *x* is the eigenvector. The DTI feature matrix is defined as Equation 6.


(6) $$D\,T\,I_{v\,e\,c\,t\,o\,r}=\left[\lambda_{1},\lambda_{2}\,\cdots \,\cdots \,\lambda_{n}\right],n\in \left[1,85\right]$$

Then, we modify the LassoNet objective function to Equation 7.


$$\begin{array}{c} m\,i\,n\,i\,m\,i\,z\,e\\ \theta ,W \end{array}\,L\,\left(\theta ,W\right)+\lambda \cdot D\,T\,I_{v\,e\,c\,t\,o\,r}\,{||\theta ||}_{1}$$

(7) $$s\,u\,b\,j\,e\,c\,t\,t\,o\,||W_{j}^{\left(1\right)}||\leq M\,\left|\theta_{j}\right|,j=1,\cdots \,d$$

So, the multi-modal LassoNet framework was constructed. We summarize the training algorithm of multi-modal LassoNet, as shown Table 2.

**TABLE 2:** Training algorithm of multi-modal LassoNet.

Algorithm: Multi-Modal LassoNet with neural network
1: **Input:** *X* ∈*R^n×*d*^* represents fMRI time series (BOLD signal), *B* represents Number of epochs, *M* represents hierarchy multiplier, ϵ represents path multiplier, α represents learning rate, *D* represents penalty matrix from DTI network. 2: **Initialize:** *L*(θ,*W*)represents the feed-forword network on the loss, λ represents the penalty, *k* represents the number of activate features, *DTI*_*V*_ = [λ_1,_λ_2,_λ_3_⋯⋯λ_*n*_ represents the multimodal matrix calculated from the penalty matrix D, *d* represents the number of features, θ ∈ ^*Rd*^represents the weights in the residual layer, *K* is the number of units in the first hidden layer, θ* and W*are the optimal parameters after iteration. 3: while k > 0 do 4: Update λ←(1+ϵ)λ*DTI*_*v*_ 5: for b ∈ (1…B) do 6: Compute gradient of the w.r.t to (θ,*W*) with back-propagation Update θ←θ−α_∇θ_⁡L*andW*←W−a_∇*W*_⁡L 7: for *j* ∈ {1…*d*} do 8: Sort the entries of $W_{j}^{\left(1\right)}$ into $\left|W_{j}^{\left(1\right)}\right|\geq \ldots \geq \left|W_{\left(j,K\right)}^{\left(1\right)}\right|$ 9: Compute $w_{n}:=\frac{M}{1+n\,M^{2}}\cdot S_{\lambda \,D}\left(|\theta_{j}|+M\cdot \sum_{i=1}^{n}|W_{\left(j,i\right)}^{\left(1\right)}|\right)$ 10: Find n*,the first *n* ∈ {0,……,*K*} such that $\left|W_{\left(j,n+1\right)}^{\left(1\right)}\right|\leq w_{n}\leq \left|W_{\left(j,n\right)}^{\left(1\right)}\right|$ 11: Update θj*←1M⋅s⁢i⁢g⁢n⁢(θj)⋅wn*,Wj(1)*←s⁢i⁢g⁢n⁢(Wj(1))⋅min⁢(wn*,|Wj(1)|) 12: end for end for 13: **return** (θ*,W(1)*) 14: end while

### Feature Detection and Model Comparison

Using the resulting images, we obtained the initial dataset of 85 participants and 187×90features in each participant. We extracted three groups from the dataset, namely, AD-HC, AD-EMCI, and EMCI-HC. For each group, the train set, validation set, and test set were selected randomly using the ratio *S*_*train*_:*S*_*valid*_:*S*_*test*_=6:2:2. Integrating with DTI structure network information, the *S*_*train*_ and *S*_*valid*_ were applied to filter the optimal λ and integrating with DTI structure network information. With the resulting λ, the *S*_*train*_ and *S*_*test*_ were used to detect features and get the sparse feature matrix that classified well in AD-HC, AD-EMCI, or EMCI-HC.

Since the multi-modal framework was optimized based on the LassoNet model, to determine the superiority of our proposed framework, we used the classic Lasso, Group Lasso, Sparse Group Lasso, and ElasticNet to compare the classification accuracy.

Given *n* data samples {(*x*_1_,*y*_1_),(*x*_2_,*y*_2_),⋯(*x*_*n*_,*y*_*n*_)},*x*_*i*_ ∈ ^*Rd*^, *x_i* was a *d* dimensional vector, that is, each observed data were composed of the values of d variables, and each *y*_*i*_ ∈ *R* was a real value. Let the mapping *f*:^*Rd*^→*R* that minimize the sum of squared errors, and the optimization objective is defined as Equation 8.


(8) $$W^{*}=a\,r\,g\,m\,i\,n_{\beta }\,\frac{1}{n}\,{||y-X\,W||}_{2}^{2}$$

The optimization objective of Lasso (Equation 8) was obtained by introducing the L1 regularization term in Equation 9.


(9) $$W^{*}=a\,r\,g\,m\,i\,n_{\beta }\,\frac{1}{n}\,{||y-X\,W||}_{2}^{2}+\lambda \,{||W||}_{1}$$

The Lasso was applied to the group and the Group Lasso was obtained as Equation 10.


(10) $$\begin{array}{c} m\,i\,n\\ W\in R^{p} \end{array}\,\left({||y-\sum_{l=1}^{L}X_{l}\,W_{l}||}_{2}^{2}+\lambda \,\sum_{l=1}^{L}\sqrt{p_{l}}\,{||W_{l}||}_{2}\right)$$

The Sparse Group Lasso was obtained by integrating the original Lasso into the Group Lasso, as Equation 11.


(11) $$\begin{array}{c} m\,i\,n\\ W\in R^{p} \end{array}\,\left({||y-\sum_{l=1}^{L}X_{l}\,W_{l}||}_{2}^{2}+\lambda_{1}\,\sum_{l=1}^{L}{||W_{l}||}_{2}+\lambda_{2}\,{||W||}_{1}\right)$$

The definition of ElasticNet was obtained by combining L1 and L2 regularization and Lasso (Equation 12).


(12) $$\begin{array}{c} m\,i\,n\\ W\in R^{p} \end{array}\,\left({||y-\sum_{l=1}^{L}X_{l}\,W_{l}||}_{2}^{2}+\lambda_{2}\,\sum_{l=1}^{L}{||W_{l}||}_{2}+\lambda_{1}\,\sum_{l=1}^{L}{||W_{l}||}_{1}\right)$$

The same *S*_*train*_ and *S*_*valid*_ were applied to filter the optimal parameters. Using the same *S*_*train*_ and *S*_*tes*_, the experiments were repeated 10 times in all five frameworks with the optimal parameters.

### Evaluation Metrics

In this study, the samples were positive and negative, and the results classified had the following cases:

True Positive (TP): the positive sample was predicted as a positive sample.
True Negative (TN): the negative sample was predicted as a negative sample.
False Positive (FP): the negative sample was predicted as a positive sample.
False Negative (FN): the positive sample was predicted as a negative sample.

ACC (accuracy) is the number of correctly classified samples divided by the total number of samples (Equation 13).


(13) $$A\,C\,C=\frac{T\,P+T\,N}{T\,P+T\,N+F\,P+F\,N}$$

SEN (sensitivity) is the proportion of pairs of all positive samples (Equation 14).


(14) $$S\,E\,N=\frac{T\,P}{T\,P+F\,N}$$

SPE (specificity) is the proportion of pairs of all negative samples (Equation 15).


(15) $$S\,P\,E=\frac{T\,N}{T\,N+F\,P}$$

GMean is the geometric mean (Equation 16).


(16) $$G\,M\,e\,a\,n=\sqrt{S\,E\,N+S\,P\,E}$$

F1 is a comprehensive evaluation indicator. Sometimes, accuracy and sensitivity needed to be considered together as Equation 17.


(17) $$F\,1=\frac{2\,T\,P}{2\,T\,P+F\,P+F\,N}$$

The receiver operating characteristic (ROC) curve and the area under curve (AUC) value are also used to evaluate the performance of the classifier.

## Results

### The Results of Parameter Optimization

**FIGURE 2:**
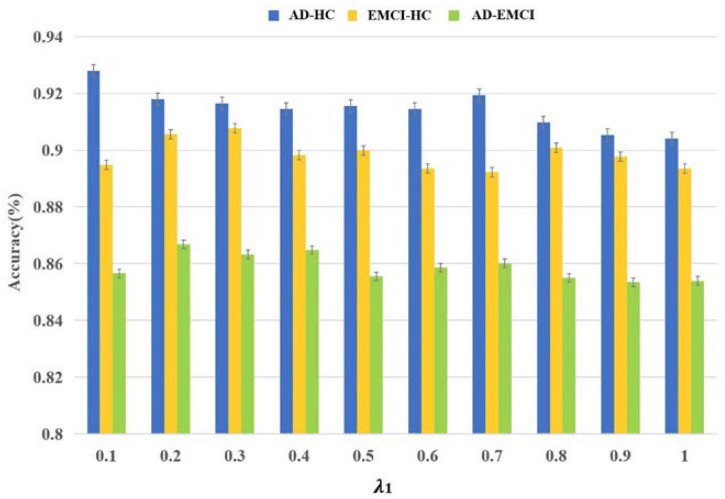
The relationship between the accuracies and λ.

Initially, 187×90=16,830 features were obtained and *S*_*train*_ and *S*_*valid*_ were applied to filter the optimal parameters. The λ was the interval of (0.1, 1), and the corresponding accuracy was calculated in each group. As shown in Figure 2, the best accuracy of the AD-HC group is 92.79% and λ is 0.1. The peak value of the EMCI-HC group is at the node of 0.3. The prediction accuracy reaches a peak with a λ value of 0.2. We can also observe that the accuracy of the AD-HC group is much higher than the other two groups. This may be caused by the large difference between AD and HC. An interesting finding is that the accuracy of the AD-EMCI group is the lowest and the gap in this group is also the lowest. This proves that the similarity between AD and EMCI is higher, and the similar features make the classification more stable.

### Comparison With Other Methods

**FIGURE 3:**
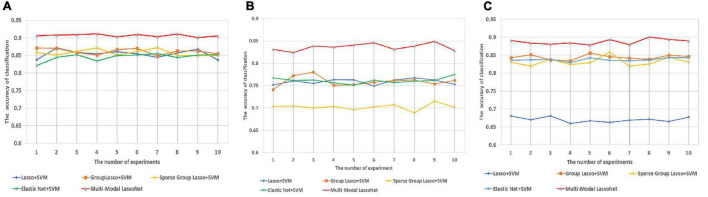
The prediction accuracy was obtained through 10 experiments for five methods in three groups. **(A)** Prediction accuracy of AD-HC group. **(B)** Prediction accuracy of AD-EMCI group. **(C)** Prediction accuracy of EMCI-HC group.

We applied the same *S*_*train*_ and *S*_*test*_ to assess the performance of the five models, and 10 independent experiments were conducted to evaluate the universality of these models. As shown in Figure 3, the Multi-modal LassoNet has good prediction accuracy, and in three groups, the accuracy of the Multi-modal LassoNet is the highest, far exceeding the other four models. The peaks of the Multi-modal LassoNet are above 90% in the AD-HC and EMCI-HC groups, and in the other four models, they are all below 90%. In the AD-EMCI group, the best accuracy is above 85%, and in the other four models, it is below 80%. Additionally, the gap of the Multi-modal LassoNet in 10 experiments is less than 2%. It can be seen from Figure 3 that the Multi-modal LassoNet framework has satisfactory classification accuracy in different groups only by adjusting the λ. The curves of the Multi-modal LassoNet also prove that the proposed framework has good stability, and the introduction of DTI information improves the classification performance of the LassoNet model.

The classification information of the five methods is presented in Table 3. Multi-modal LassoNet classifiers reported very good performance. In the AD-HC group classification, ACC, SEN, SPE, GMean, and F1 were 90.68 ± 0.34, 88.81 ± 0.68, 91.91 ± 0.55, 90.34 ± 0.36, and 88.25 ± 0.52, respectively. In the AD-EMCI group classification, ACC, SEN, SPE, GMean, and F1 were 83.63 ± 0.74, 87.32 ± 1.22, 79.00 ± 1.33, 83.05 ± 0.76, and 85.70 ± 0.84, respectively. In the EMCI-HC group classification, ACC, SEN, SPE, GMean, and F1 were 88.77 ± 0.70, 90.87 ± 1.05, 87.06 ± 0.95, 88.94 ± 0.69, and 87.92 ± 0.83, respectively.

**TABLE 3:** The classification performance comparison of the five methods.

Group	Methods	ACC (%) ± SD	SEN (%) ± SD	SPE (%) ± SD	GMean (%) ± SD	F1 (%) ± SD
AD-HC	Lasso + SVM	85.45 ± 1.10	75.34 ± 2.87	91.89 ± 0.81	83.19 ± 1.49	80.05 ± 1.66
	GroupLasso + SVM	86.16 ± 0.78	77.13 ± 1.73	91.97 ± 1.00	84.24 ± 0.93	80.75 ± 2.75
	ElasticNet + SVM	84.56 ± 1.00	74.90 ± 2.14	90.72 ± 1.24	82.41 ± 1.13	79.04 ± 1.24
	Sparse Group Lasso + SVM	85.75 ± 0.83	75.99 ± 2.29	**92.20 ± 0.88**	83.69 ± 1.11	80.89 ± 1.18
	Multi-modal LassoNet	**90.68 ± 0.34**	**88.81 ± 0.68**	91.91 ± 0.55	**90.34 ± 0.36**	**88.25 ± 0.52**
AD-EMCI	Lasso + SVM	75.88 ± 0.58	93.06 ± 0.87	54.22 ± 0.95	71.03 ± 0.61	81.15 ± 0.56
	GroupLasso + SVM	75.92 ± 1.04	**93.12 ± 0.63**	54.33 ± 1.77	71.12 ± 1.14	81.16 ± 0.87
	ElasticNet + SVM	76.13 ± 0.61	92.91 ± 0.94	54.98 ± 1.96	71.45 ± 1.01	81.27 ± 0.60
	Sparse Group Lasso + SVM	70.23 ± 0.63	90.44 ± 1.05	44.69 ± 1.82	63.56 ± 1.04	77.05 ± 0.67
	Multi-modal LassoNet	**83.63 ± 0.74**	87.32 ± 1.22	**79.00 ± 1.33**	**83.05 ± 0.76**	**85.70 ± 0.84**
EMCI-HC	Lasso + SVM	67.04 ± 0.69	78.67 ± 1.98	57.61 ± 1.49	67.30 ± 0.68	68.12 ± 0.90
	GroupLasso + SVM	84.42 ± 0.65	**96.62 ± 0.65**	74.43 ± 0.98	84.80 ± 0.52	84.08 ± 0.57
	ElasticNet + SVM	83.76 ± 0.42	94.69 ± 0.69	74.72 ± 0.92	84.11 ± 0.44	84.07 ± 0.41
	Sparse Group Lasso + SVM	83.20 ± 1.15	95.83 ± 0.88	72.74 ± 1.43	83.49 ± 1.14	70.86 ± 1.16
	Multi-modal LassoNet	**88.77 ± 0.70**	90.87 ± 1.05	**87.06 ± 0.95**	**88.94 ± 0.69**	**87.92 ± 0.83**

**FIGURE 4:**
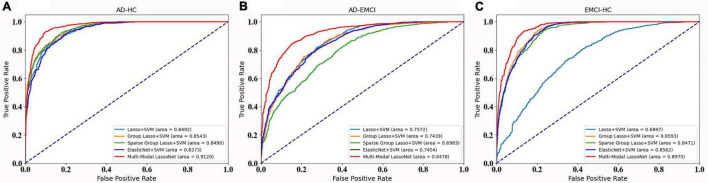
The ROC curve of the five methods in three groups. **(A)** Prediction accuracy of AD-HC group. **(B)** Prediction accuracy of AD-EMCI group. **(C)** Prediction accuracy of EMCI-HC group.

For further validation of our framework and results, we plot the ROC curves of five methods for the AD-HC, AD-EMCI, and EMCI-HC groups, as shown Figure 4. The AUC values of our proposed Multi-modal LassNet for AD-HC, AD-EMCI, and EMCI-HC groups were 0.9120, 0.8478, and 0.8975, respectively.

## Discussion

Modeling techniques based on a single neuroimaging modality lacked the spatial and temporal high-resolution information brought by different modalities in characterizing the brain network structure, and could not fully reflect the dynamic mechanism of brain network connections (Tulay et al., 2019; Zhuang et al., 2019; Lei et al., 2020). Therefore, we proposed a multi-modal LassoNet model that was a Lasso neural network modeling framework using multi-modal information fusion. This method fused two modalities of fMRI and DTI in a sparse Lasso neural network framework and introduced connection strength and subject structure to complete the construction of a multi-modal brain network. Our proposed method mainly addresses two issues, which include the selection of AD-related brain ROIs and the classification and diagnosis of AD. The experimental results showed that the multi-LassoNet modeling of multi-modal information could facilitate higher sensitivity of disease diagnosis and effectively improved the accuracy of model classification. The good classification performance also revealed that the detected features of the multi-modal model based on fMRI and DTI reflected that the brain atrophy caused by the disease process would lead to the decrease of white matter fiber connectivity (Gupta et al., 2020). It also proved that structural connectivity and functional brain network features between connections had coupling effects.

Compared with the current popular Lasso method, Group Lasso, Sparse Lasso, and elastic network method, it was proved that the proposed multi-modal Lasso-based neural network method was higher than other methods in classification performance and had strong regularization parameter stability. It proved that fusion of multi-modal information more effectively identified brain network features. Moreover, the results indicated that the constraint effect of the DTI structural network and the introduction of the strength of the brain area connection had a certain degree of influence on the validity of the multi-modal brain network model. Table 4 shows the top 15 important brain regions with different classification results.

**TABLE 4:** Discriminative brain regions.

Group	ID	Regions	Abbreviation	ID	Regions	Abbreviation
AD-HC	61	Parietal_Inf_L	IPL.L	19	Supp_Motor_Area_L	SMA.L
	24	Frontal_Sup_Medial_R	SFGmed.R	59	Parietal_Sup_L	SPG.L
	37	Hippocampus_L	HIP.L	83	Temporal_Pole_Sup_L	TPOsup.L
	79	Heschl_L	HES.L	64	SupraMarginal_R	SMG.R
	7	Frontal_Mid_L	MFG.L	81	Temporal_Sup_L	STG.L
	73	Putamen_L	PUT.L	52	Occipital_Mid_R	MOG.R
	15	Frontal_Inf_Orb_L	ORBinf.L	32	Cingulum_Ant_R	ACG.R
	56	Fusiform_R	PoCG.L			
EMCI-HC	37	Hippocampus_L	HIP.L	14	Frontal_Inf_Tri_R	IFGtriang.R
	27	Rectus_L	REC.L	59	Parietal_Sup_L	SPG.L
	17	Rolandic_Oper_L	ROL.L	88	Temporal_Pole_Mid_R	TPOmid.R
	30	Insula_R	INS.R	44	Calcarine_R	CAL.R
	6	Frontal_Sup_Orb_R	ORBsup.R	49	Occipital_Sup_L	SOG.L
	8	Frontal_Mid_R	MFG.R	31	Cingulum_Ant_L	ACG.L
	38	Hippocampus_R	HIP.R	7	Frontal_Mid_L	MFG.L
	15	Frontal_Inf_Orb_L	ORBinf.L			
AD-EMCI	22	Olfactory_R	OLF.R	63	SupraMarginal_L	SMG.L
	32	Cingulum_Ant_R	ACG.R	57	Postcentral_L	PoCG.L
	89	Temporal_Inf_L	ITG.L	51	Occipital_Mid_L	MOG.L
	82	Temporal_Sup_R	STG.R	24	Frontal_Sup_Medial_R	SFGmed.R
	85	Temporal_Mid_L	MTG.L	39	ParaHippocampal_L	PHG.L
	42	Amygdala_R	AMYG.R	13	Frontal_Inf_Tri_L	IFGtriang.L
	28	Rectus_R	REC.R	60	Parietal_Sup_R	SPG.R
	8	Frontal_Mid_R	MFG.R			

**FIGURE 5:**
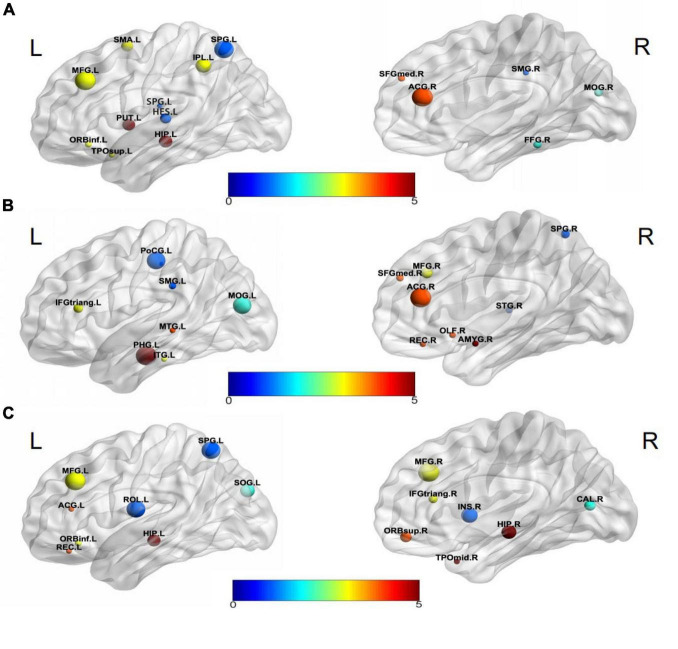
Visualization of discriminative brain regions. **(A)** AD-HC, **(B)** AD-EMCI, and **(C)** EMCI-HC.

Visualization selected discriminative brain regions using the BrainNet Viewer toolbox (Xia et al., 2013), as shown in Figure 5. By analyzing the brain regions classifying AD-HC,AD-EMCI, and EMCI-HC, we found that the brain regions belonging to Hippocampus, Frontal_Inf_Orb_L, and Parietal_Sup_L were among the top 15 brain regions. Previous studies had found that the hippocampus of the brain was responsible for human memory and spatial activities and was closely related to AD pathology (Douaud et al., 2011; Fares et al., 2019). In addition, some studies had also shown that functional atrophy in the parahippocampal gyrus is an early marker of AD/MCI disease (Wang et al., 2016), and the parahippocampal gyrus shows a more distinct ability than the hippocampus in the early stage of the disease (Zhu et al., 2019). Frontal_Inf_Orb_L corresponded to the region of interest recommended by physicians for the clinical diagnosis of AD (Jiang et al., 2015). Parietal_Sup_L may be associated with the underlying mechanism of its clinical effect, and it may play a role in the potential compensatory mechanism of mobilizing more regions to complete the function after a functional decline (He et al., 2021). The Hippocampus_L and Hippocampus_R found in the AD-HC and EMCI-HC groups were reported as the pathogenic regions of AD. Chik et al. (Yuan et al., 2022) found that the neurosteroids in the hippocampus were changed during the progression of Lv et al. (2022) found that compared to the healthy mouse, the mice having TYRO protein kinase-binding protein had insufficient learning and memory abilities, and the amyloid β in the hippocampus was increased, which worsened with aging. Liu et al. (2022) proved that memory could be improved by enhancing the functional activity in the hippocampus and the medial prefrontal cortex. Moreover, the hippocampus region was not found in AD-EMCI. This gives a message that the difference in the hippocampus between AD and EMCI is not obvious, and their main difference is found in other brain regions, such as the Amygdala_R, which is not found in the other two groups. Hong et al. (2022) reported tau deposition in the parahippocampus and amygdala by studying positron emission tomography (PET) images in patients with AD. The amygdala atrophy was found in mild AD subjects and could be used to predict the Mini-Mental State Examination scores and hippocampal atrophy (Poulin et al., 2011).

In addition, the Putamen_L was reported to be the earliest brain region to show increased Aβ deposition and is a marker of cognitive decline (Zammit et al., 2020; Cogswell et al., 2021). The Fusiform_R was confirmed to be a characteristic region of AD (Guo et al., 2017; Sprung et al., 2021). Brain network analysis results generally had a high sensitivity to segmentation template selection. Different segmentation templates produced different brain network topology structures, which might potentially affect the reproducibility of model classification performance. The segmentation template used in this paper was the AAL structure of 90 brain regions. However, in the future, the robustness value of the proposed method would be further verified from the perspectives of multiple segmentation scales.

In this study, a deterministic fiber tracking technique derived from DTI images was used to construct a structural brain network in a multi-modal modeling framework. But this tracking method only considered the trajectories where white matter fibers cross or diverge (Lei et al., 2021). Therefore, there may be biases in determining the most reasonable fiber configuration, affecting the accuracy of structural network construction. Future research work will consider adopting a more efficient probabilistic fiber tract-tracing strategy to obtain the probability value of brain area connection to complete accurate multi-modal brain network construction.

In this study, we proposed a novel multi-modal LassoNet framework for the discriminant analysis of features. This research is an attempt to apply fMRI and DTI multi-modal information and sparse representation technology to the research of neural network framework, and provides a new idea for designing a brain network modeling framework that integrates more modal information in the future. The features of multi-modal data can be fused to obtain more comprehensive pathological information. Compared to the conventional methods, the proposed method seeks to identify AD-related brain ROIs and in the classification and diagnosis of AD. The high-performance classification implied that the proposed multi-modal LassoNet framework was beneficial for the early diagnosis and prediction of AD disease.

## Data Availability Statement

Publicly available datasets were analyzed in this study. Data used in preparation of this article were obtained from the Alzheimer’s Disease Neuroimaging Initiative (ADNI) database (adni.loni.usc.edu). The data is available at http://adni.loni.usc.edu/.

## Ethics Statement

Ethical review and approval was not required for the study on human participants. Data collection and sharing for this project were obtained from the Alzheimer’s Disease Neuroimaging Initiative (ADNI).

## Author Contributions

XM, CB, WL, and ZJ led and supervised the research. XM, WL, and ZJ designed the research and wrote the article. XF, QW, and ZW performed data preprocessing. XM, JL, WL, and ZJ performed feature extraction and selection by the multi-modal LassoNnet framework. XF, QW, and CB did a discriminative analysis of brain regions. All authors contributed to the article and approved the submitted version.

## Conflict of Interest

The authors declare that the research was conducted in the absence of any commercial or financial relationships that could be construed as a potential conflict of interest.

## Publisher’s Note

All claims expressed in this article are solely those of the authors and do not necessarily represent those of their affiliated organizations, or those of the publisher, the editors and the reviewers. Any product that may be evaluated in this article, or claim that may be made by its manufacturer, is not guaranteed or endorsed by the publisher.

## Footnotes

1
www.adni-info.org

2
https://www.fil.ion.ucl.ac.uk/spm/software/spm12/

3
http://rfmri.org/DPABI

4
https://fsl.fmrib.ox.ac.uk/fsl/fslwiki/

5
https://www.nitrc.org/frs/?group_id=582
